# Detection of *Entamoeba histolytica* DNA in the Saliva of Amoebic Liver Abscess Patients Who Received Prior Treatment with Metronidazole

**DOI:** 10.3329/jhpn.v26i4.1883

**Published:** 2008-12

**Authors:** Krishna Khairnar, Subhash Chandra Parija

**Affiliations:** Department of Microbiology, Jawaharlal Institute of Postgraduate Medical Education and Research, Puducherry 605 006, India

**Keywords:** Amoebiasis, DNA, *Entamoeba histolytica*, Metronidazole, Polymerase chain reaction, Saliva, India

## Abstract

Saliva is an easily-accessible and a non-invasive clinical specimen alternate to blood and liver pus. An attempt was made to detect *Entamoeba histolytica* DNA released in the saliva of amoebic liver abscess (ALA) patients by applying 16S-like rRNA gene-based nested multiplex polymerase chain reaction (NM-PCR). The NM-PCR detected *E. histolytica* DNA in the saliva of eight (28.6%) of 28 ALA patients. The NM-PCR result was negative for *E. histolytica* DNA in the saliva of all the eight ALA patients who were tested prior to treatment with metronidazole but was positive in the saliva of eight (40%) of 20 ALA patient who were tested after therapy with metronidazole. The NM-PCR detected *E. histolytica* DNA in liver abscess pus of all 28 (100%) patients with ALA. The TechLab *E. histolytica* II enzyme-linked immunosorbent assay was positive for *E. histolytica* Gal/GalNAc lectin antigen in the liver abscess pus of 13 (46.4%) of the 28 ALA patients. The indirect haemagglutination (IHA) test was positive for anti-amoebic antibodies in the serum of 22 (78.6%) of the 28 ALA patients and 2 (5.7%) of 35 healthy controls. The present study, for the first time, demonstrates the release of *E. histolytica* DNA in the saliva of ALA patients by applying NM-PCR.

## INTRODUCTION

The use of saliva as a diagnostic fluid has been increasingly reported worldwide in the last decade. Technological advancement has taken place during the past few years enabling the use of saliva as a clinical specimen to diagnose disease and predict disease progression (1).

Initially, saliva was used as a clinical specimen for antibody detection in the diagnosis of infectious diseases. Detection of salivary antibody was found to be useful for the diagnosis of bacterial infections caused by *Helicobacter pylori, Shigella*, and *Borrelia burgdorferi* (2-4) and various viral infections, such as hepatitis A, hepatitis B, hepatitis C, measles, mumps, rubella, rotavirus, dengue, parvovirus B 19, and HIV (5-11). Detection of salivary antibody has also been studied for the diagnosis of some parasitic infections caused by *Toxoplasma gondii, Schistosoma mansoni, Taenia solium*, and *Entamoeba histolytica* (12-15).

Subsequently, saliva has also been used for the detection of antigen in the diagnosis of pneumococcal pneumonia (16), hepatitis B virus, measles, mumps, and rubella (17-20). There is only one report till date on the detection of salivary lectin antigen of *E. histolytica* for the diagnosis of amoebic liver abscess (ALA) with a sensitivity and specificity of 22% and 97.4% respectively (21).

The reports on the use of saliva for the detection of DNA for the diagnosis of infectious diseases, however, are limited (22-26). The polymerase chain reaction (PCR) has been used for facilitating diagnosis of viral infections, such as Epstein-Barr, cytomegalovirus, human herpes virus 6, 7, and 8, and rabies using saliva (22-25). The PCR has also been evaluated for the detection of *H. pylori-*associated peptic ulcer, by demonstration of *H. pylori* DNA in saliva (26). However, reports on the detection of DNA in saliva of patients with parasitic infection, even amoebiasis, is still lacking. In the present study, we, therefore, made an attempt to detect *E. histolytica* DNA, possibly released in the saliva of ALA patients by applying a 16S-like rRNA gene-based nested multiplex PCR (NM-PCR) assay. ALA is a condition which is the most important and serious extra-intestinal manifestation of amoebiasis, which is associated with high morbidity and mortality. An early and specific diagnosis of the condition followed by immediate treatment reduces morbidity and mortality due to the disease to a great extent.

## MATERIALS AND METHODS

### 

#### Sample details

The present study was conducted in the Jawaharlal Institute of Postgraduate Medical Education and Research (JIPMER) Hospital, Puducherry, India, during August 2005–March 2006.

**Patients with ALA (n=28):** The study included 28 ALA patients; diagnosis was done on the basis of radiological, symptomatological and laboratory criteria (27,28), such as: (a) ultrasonography revealing a space-occupying lesion in the liver suggestive of an abscess; (b) clinical symptoms, such as pain in the right hypochondrium, lower chest, back, or tip of the right shoulder, and fever; (c) distended and/or tender liver, generally without jaundice; (d) chest radiograph showing raised right dome of the diaphragm; (e) treatment with anti-amoebic drugs, e.g. metronidazole, results in improvement of the condition; (f) positive indirect haemagglutination (IHA) of serum antibody showing a titre (≥1:128) against *E. histolytica*; and (g) liver aspirate appeared like anchovy sauce but was bacteriologically sterile.

In the present study, the 28 ALA patients included eight patients on whom the metronidazole therapy was not initiated and 20 patients on whom the metronidazole therapy was already initiated.

**Patients with pyogenic liver abscess (PLA) and other diseases of the liver (n=21):** The study included cases of PLA (n=13), hydatid cyst in liver (n=2), liver hepatoma (n=1), liver cirrhosis (n=3), and viral hepatitis (n=2).

**Healthy control (n=35):** The study included 35 healthy controls who had no history of recent dysentery or diarrhoea and whose stool samples were negative for *E. histolytica*-associated infection by microscopy and culture.

#### Sample collection

**Saliva:** Saliva specimens were collected from all the 28 ALA patients, 21 patients with PLA and other diseases of the liver, and 35 healthy controls. 5 mL of saliva specimen was collected from each individual in a sterile container using the aseptic techniques and was stored at 4 °C until used.

**Liver abscess pus:** The aspiration of liver abscess pus was indicated only under the following conditions (27): (a) to rule out a pyogenic abscess; (b) the failure to respond clinically in 3-5 days; (c) the threat of imminent rupture; and (d) the prevention of rupture of left-lobe abscess into the pericardium. The liver abscess pus aspirates were performed, only for clinical purposes as judged by the clinicians for the patient care and not for the purpose of this study. Liver abscess pus was obtained under ultrasound guidance from all the 28 ALA patients and 13 PLA patients and was stored at −20 °C in a sterile container until used.

**Blood:** Blood specimens were collected from all the 28 ALA patients, 21 with PLA and other diseases of the liver, and 35 healthy controls. 5 mL of venous blood was collected in a sterile container; sera were separated and stored at −20 °C until used.

#### Detection of anti-amoebic antibodies in serum by IHA

The Rapid-IHA was performed on serum specimen as per the method described earlier (29). A titre of ≥1:128 was considered to be positive for ALA (30).

#### Detection of Gal/GalNAc lectin antigen in liver pus by TechLab *E. histolytica* II ELISA

The TechLab *E. histolytica* II test was performed on liver abscess pus specimens to detect *E. histolytica*-specific Gal/GalNAc lectin antigen as per the method described earlier (28).

### NM-PCR

#### *Extraction of* Entamoeba *genomic DNA from saliva and liver abscess pus*

The protocol for extraction of DNA from saliva and liver abscess pus specimen has been modified in our laboratory from cetyltrimethylammonium bromide (CTAB) DNA extraction protocol originally described for DNA extraction from amoebic culture (31).

**Saliva:** Briefly, 5 mL of saliva was centrifuged at 12,000 g for eight minutes at 4 °C. The supernatant was discarded, and the pellet was suspended in 250 μL of sterile distilled water. To the suspension 5 μL of proteinase-K (10 mg/mL) and 40 μL of 10% sodium dodecyl sulphate was added and incubated for three hours at 65 °C. Then, 60 μL of 5 M sodium chloride and 15 μL of 10% CTAB were added to the mixture and incubated for 45 minutes at 65 °C. This was followed by extractions with equal volumes of chloroform and then phenol-chloroform-isoamyl alcohol. The DNA was precipitated with ice-cold ethanol. The dried DNA pellet was dissolved in 50 μL of sterile distilled water.

**Liver abscess pus:** The extraction of *Entamoeba* genomic DNA from liver abscess pus was performed as per the method described earlier (32).

The extracted DNA from saliva and liver abscess pus sample was passed through DNA clean-up spin columns (Bangalore Genei KT-62, Bangalore). The DNA was stored at −20 °C until used.

#### Quantification of DNA in saliva and liver abscess pus

DNA quantification in spin column-purified DNA extract from saliva and liver abscess pus specimens was determined by ultraviolet (UV) absorbance using a Cintra 5 double beam spectrophotometer. DNA yield was calculated on the basis of UV absorbance × dilution. The purity of the nucleic acid in the samples was estimated by the ratio of readings at 260 nm and 280 nm (OD260/OD280).

#### Primers used

Based on the sequences of the 16S-like rRNA gene of *E. histolytica, E. dispar,* and *E. moshkovskii* nested set of primers (designated E-1/E-2, EH-1/EH-2, ED-1/ED-2, and Mos-1/Mos-2) were used as previously described (32).

In addition, we have used an internal amplification control (IAC) targeting human 18S ribosomal RNA gene to rule out false-negative results in clinical specimens as previously described (32).

#### Primer validation

The primer sequences for *E. moshkovskii, E. histolyti-ca, E. dispar*, and human IAC were subjected to the basic local alignment search tool (BLAST) in the genome database of all organisms available at http://www.ncbi.nlm.nih.gov/blast/ and were found to be specific for the study. The 439 bp PCR products of *E. histolytica* species from representative saliva and liver abscess pus specimens were confirmed by getting both the strands of DNA sequenced on ABI3730XL sequencer (Macrogen, Seoul, South Korea). The nucleotide sequencing was done using species-specific primers, i.e. EH-1/EH-2, for *E. histolytica*. The nucleotide sequences were analyzed for homology using the nucleotide-nucleotide BLAST search feature available at http://www.ncbi.nlm.nih.gov/blast/. The identities between the nucleotide sequencing result of representative 439 bp PCR products of *E. histolytica* species from saliva and liver abscess pus specimens with the sequence deposited in GenBank [accession number: X56991] were analyzed using ‘Align two sequences (bl2seq)’ feature available at http://www.ncbi.nlm.nih.gov/blast/. The multiple sequence alignment of the nucleotide sequences of representative 439 bp PCR productsof *E. histolytica* species from saliva and liver abscess pus specimens with the sequence deposited in GenBank [accession number: X56991] was also done using the BioEdit software (version 7).

#### Standard strains

Three standard strains—*E. histolytica* HM-1:IMSS, *E. dispar* SAW760, and *E. moshkovskii* Laredo—were used as positive control in the present study. The lyophilized DNA of these strains was generously gifted by Dr. C. Graham Clark from the London School of Hygiene & Tropical Medicine, UK.

#### NM-PCR for saliva and liver abscess pus

**Saliva PCR:** For a reaction volume of 25 μL, comprising 2.5 μL of 10X PCR buffer (Biogene, Kimbolton, UK), 1.0 μL of 25 mM MgCl2 (Bangalore Genei, Bangalore, India), 0.75 μL of deoxy-ribonucleotide triphosphate mix (10 mM each dNTP, Biogene, Kimbolton, UK), 0.3 μL (5 IU/μL) of *Taq* polymerase (Biogene, Kimbolton, UK), 10 picomoles of target DNA primers (IDT, Coralville, USA), and 5 picomoles of IAC primers (IDT, Coralville, USA) were added in genus- and species-specific PCR. The template DNA volume was 2 μL for both genus- and species-specific PCR. The PCR tubes were finally placed in an Eppendorf thermal cycler (master cycler gradient; Westbury, NY, USA).

The conditions for genus-specific PCR were as follows; the PCR mix was subjected to an initial denaturation at 96 °C for two minutes, followed by 30 cycles—each consisting of 92 °C for 60 seconds (denaturation), 56 °C for 60 seconds (annealing), and 72 °C for 90 seconds (extension). Finally, one cycle of extension at 72 °C for seven minutes was performed. In the species-specific NM-PCR (which had multiple primer-sets in the same tube), only the annealing temperature was changed to 48 °C, leaving the other parameters of the amplification cycles unchanged.

**Liver abscess pus PCR:** The PCR mix composition and PCR conditions for the amplification of *Entamoeba* 16S-like rRNA gene from liver abscess pus was performed as per the method described earlier (32).

3.5 μL of the amplification product was separated by electrophoresis through 1.8% agarose gel (Agarose Low EEO; Bangalore Genie, Bangalore, India) containing ethidium bromide in 0.5 X Tris-borate-EDTA at 120 V for 45 minutes and was visualized under UV light for bands of DNA of appropriate sizes (Fig.).

**Fig.**
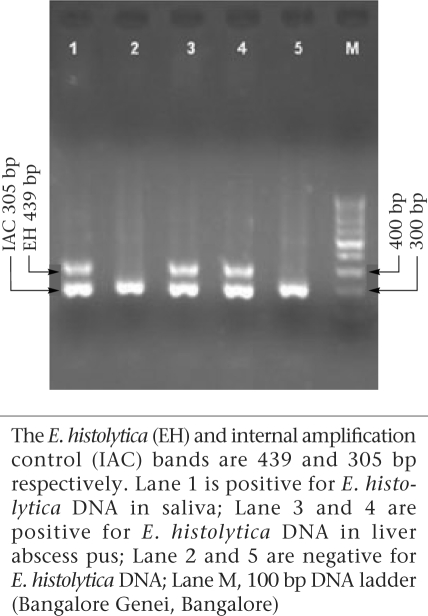
Results of nested multiplex PCR on repre- sentative saliva and liver abscess pus specimen

#### Analysis of statistical data

Sensitivity was calculated as the number of patients with positive test results/total number of patients × 100. Specificity was calculated as the number of controls with negative test results divided by total number of controls × 100. To calculate the significance of the difference in sensitivities, McNemar's chi-square test was applied. The McNemar's test was performed using the Graph Pad Software.

## RESULTS

### 

#### Detection of anti-amoebic antibodies in serum by IHA

The IHA test was positive for anti-amoebic antibo-dies in the serum of 22 (78.6%) of the 28 ALA patients and two (5.7%) of 35 healthy controls. The IHA test was negative for anti-amoebic antibodies in the serum of all 21 patients with PLA and other diseases of the liver.

Detection of Gal/GalNAc lectin antigen in liver pus by TechLab

#### *E. histolytica* II ELISA

The TechLab *E. histolytica* II enzyme-linked immunosorbent assay (ELISA) test was positive for *E. histolytica* Gal/GalNAc lectin antigen in the liver abscess pus of 13 (46.4%) of the 28 ALA patients. The TechLab *E. histolytica* II ELISA detected *E. histolytica* lectin antigen in the liver pus of all the eight (100%) ALA patients who were tested prior to treatment with metronidazole but was detected in only five (25%) of the 20 ALA patients who were tested after the initiation of therapy with metronidazole. This might be due to the rapid clearing of amoebic antigen from the liver pus due to killing of *E. histolytica* trophozoites on treatment with metronidazole. The TechLab *E. histolytica* II ELISA test was negative for *E. histolytica* Gal/GalNAc lectin antigen in the liver abscess pus of all the 13 patients with PLA.

### NM-PCR

#### Quantification of DNA in saliva and liver abscess pus

The average DNA yield for all saliva and liver abscess pus specimens was found to be approximately 33 and 85 μg/mL respectively by spectrophotometric analysis. The purity of DNA extract from saliva and liver abscess pus specimens was found to be satisfactory as the ratio of readings at 260 nm and 280 nm (OD260/OD280) was approximately 1.8.

#### Primer validation

The sequencing result of representative 439 bp PCR products of *E. histolytica* species from saliva and liver abscess pus specimens showed 99-100% identities to the sequence deposited in GenBank [accession number: X56991]. The multiple sequence alignment of the nucleotide sequences of representative 439 bp PCR products of *E. histolytica* species from saliva and liver abscess pus specimens with the sequence deposited in GenBank [accession number: X56991] showed no discrepancy.

#### NM-PCR for saliva and liver abscess pus

The NM-PCR performed on the representative saliva specimen is shown in the figure. The NM-PCR detected *E. histolytica* DNA in eight (28.6%) of the 28 saliva specimens collected from the ALA patients (Table). The NM-PCR was negative for *E. histolytica* DNA in the saliva of all the eight ALA patients who were tested prior to treatment with metronidazole but was positive in the saliva of eight (40%) of the 20 ALA patients who were tested after therapy with metronidazole. The NM-PCR was negative for *E. histolytica* DNA in the saliva of all the 21 patients with PLA and other diseases of the liver and 35 healthy controls (Table). In this study, the probability of negative NM-PCR results in the saliva specimens due to PCR inhibitors was ruled out by the inclusion of an IAC in the PCR reaction.

**Table.** Detection of *Entamoeba* DNA in saliva and liver abscess pus specimens of ALA patients by applying 16S-like rRNA gene-based nested multiplex PCR

Diagnosis	No. of patients	No. (%) of positive results by					
		16S-like rRNA gene-based nested multiplex PCR for					
		Saliva specimens			Liver abscess pus specimens		
		*E. histolytica*	*E. dispar*	*E. moshkovskii*	*E. histolytica*	*E. dispar*	*E. moshkovskii*
Amoebic liver abscess	28	8 (28.6)	0	0	28 (100)	0	0
Pyogenic liver abscess	13	0	0	0	0	0	0
Other diseases of liver	8	0	0	0	NA	NA	NA
Healthy controls	35	0	0	0	NA	NA	NA

NA=Not applicable

The result of NM-PCR performed on the liver abscess pus is depicted in the figure. The NM-PCR was positive for *E. histolytica* DNA in all the 28 liver abscess pus specimens (100%) from the ALA patients (Table). The test did not detect *E. histolytica* DNA in liver abscess pus from all the 13 patients with PLA.

## DISCUSSION

It is difficult to differentiate clinically the ALA from PLA and from other space-occupying lesions of the liver, such as hydatid cyst and liver hepatoma (27,33). Although the imaging techniques are highly sensitive to detect abscesses in the liver of varied aetiology, these fail to distinguish specifically ALA from that of PLA.

The demonstration of *E. histolytica* trophozoite in the liver pus by microscopy confirms the diagnosis of ALA, but in best of the conditions, the amoebic trophozoites can be demonstrated in only 15% of liver pus (34). Therefore, the laboratory diagnosis of ALA is usually established by antibody-based serological tests, but the serum antibody levels in individuals living in endemic areas continue to remain positive even for years after eradication of infection with *E. histolytica* (35-37). A monoclonal antibody-based second-generation TechLab *E. histolytica* II ELISA has been reported to be 78% and 40.7% sensitive for the detection of *E. histolytica* Gal/GalNAc lectin antigen in the serum and liver pus of ALA patients respectively (28).

Earlier, the PCR to detect *Entamoeba* DNA in stool samples has been evaluated as a sensitive and specific method for the diagnosis of intestinal amoebiasis (38-46). Studies conducted in various laboratories worldwide have shown that PCR has a varied sensitivity ranging from 33% to 100% for detecting *E. histolytica* DNA in liver abscess pus for the diagnosis of ALA (28,32,47-49). Recently, we have reported, for the first time, the excretion of *E. histolytica* DNA in the urine of ALA patients by applying PCR (32).

Like urine, saliva is also an easily-accessible and non-invasive body-fluid. Detection of salivary antigen has been studied earlier for the diagnosis of a few viral infections, such as Epstein-Barr virus and hepatitis B virus (17,50). There is only one report on the detection of *E. histolytica* Gal/GalNAc lectin antigen in the saliva of ALA patients (21). Although reports are available for the detection of salivary DNA by PCR for the diagnosis of Epstein-Barr virus, cytomegalovirus, human herpes virus 6, 7, and 8, rabies virus, and *H. pylori* (22-26), till now, to the best of our knowledge, there is no report available on the detection of *E. histolytica* DNA in saliva.

In the present study, an attempt was made, for the first time, to detect *Entamoeba* DNA in saliva of ALA patients by applying NM-PCR. The NM-PCR in the study detected *E. histolytica* DNA in eight (28.6%) of the 28 saliva specimens collected from the ALA patients (Table). The assay detected *E. histolytica* DNA in saliva of eight of the 20 cases with ALA, who were already treated with metronidazole. The PCR did not detect *E. histolytica* DNA in saliva of the remaining eight cases of ALA, who were not treated with metronidazole. This might be due to the release of *E. histolytica* DNA from the dying *E. histolytica* trophozoites when metronidazole therapy was initiated, which might have led to the release of *E. histolytica* DNA in the saliva of ALA patients.

The sensitivity of NM-PCR to detect *E. histolytica* DNA in the saliva was 28.6%. This was found to be significantly lower than the sensitivity of NM-PCR to detect *E. histolytica* DNA in the liver abscess pus (100%), using McNemar's chi-square test (p<0.0001).

The NM-PCR was negative for *E. histolytica* DNA in the saliva of all the 21 patients with PLA and other diseases of the liver, and 35 healthy controls. This represents a specificity of 100% (Table). In the present study, none of the saliva and liver abscess pus specimens was positive for either *E. dispar* or *E. moshkovskii* by the NM-PCR, which confirms the non-invasive nature of these non-pathogenic species (Table).

The present study, for the first time, demonstrates the release of *E. histolytica* DNA in the saliva specimens of ALA patients by applying NM-PCR. However, as a diagnostic tool, the limitation of NM-PCR was that it could only detect *E. histolytica* DNA in the saliva specimens of ALA patients who had already received treatment with metronidazole with a sensitivity of only 40% but could not detect *E. histolytica* DNA in the saliva specimens of untreated ALA patients. Therefore, the present study on the detection of *E. histolytica* DNA in the saliva of ALA patients may not be used as a diagnostic tool. Nevertheless, the saliva as a clinical specimen offers distinctive advantage over blood as a diagnostic fluid. The first and foremost advantage is that it can be collected non-invasively by individuals with minimum training. Second, the use of saliva as a clinical specimen may prove to be a cost-effective approach for the screening of a large number of patients, especially in epidemiological studies. Finally, saliva has the potential to be used for the diagnosis of invasive infectious diseases because it is collected readily and is also known to contain serum constituents. The serum constituents present in saliva are derived from the salivary gland's local vasculature which ultimately reaches the oral cavity through the flow of gingival fluid (51). However, we feel that additional studies are needed in the field before the true clinical and diagnostic value of saliva as a clinical specimen can be established.

## ACKNOWLEDGEMENTS

The authors thank Dr. C. Graham Clark from the London School of Hygiene & Tropical Medicine for providing them with lyophilized DNA of standard cultures of *E. histolytica* HM-1:IMSS, *E. dispar* SAW760, and *E. moshkovskii* Laredo. The authors also thank Dr. K.V. Kirubasankar, Department of Preventive and Social Medicine, Jawaharlal Institute of Postgraduate Medical Education and Research, Puducherry, India, for analyzing statistical data.
